# 210 Surveillance of Gram-Negative Bacteria in Pediatric Patients Requiring Mechanical Ventilation Using Whole-Genome Sequencing

**DOI:** 10.1017/ash.2026.10595

**Published:** 2026-06-23

**Authors:** Matthew Gosen, Naomi Hauser

**Affiliations:** 1 UC Davis

## Abstract

**Introduction:** The BioFire® FilmArray® meningitis/encephalitispanel can be a helpful test for early diagnosis of community acquired central nervous system infection. However, excessive ordering can lead to patient harm, increased healthcare costs, and excessive environmental impact. Despite a diagnostic stewardship algorithm in place at our institution, ME panels are frequently nondiagnostic, prompting inquiry into whether our diagnostic stewardship algorithm could be improved and if a ME panel ordering decision rule could improve test utility. Objective: Review ME panels performed over a one month period and evaluate for adherence to our institution’s diagnostic stewardship pathway. **Methods:** We performed a retrospectivechart review of ME panels performed on adult patients over a one month time period at our institutionfor order indication,progress note clinical indication, other cerebrospinal fluid (CSF) test results, ME panel result, and patient outcome. Adherence to our current microbiology lab diagnostic stewardship algorithm and clinical utility were evaluated. The current algorithm calls for ME panels to be performed only on CSF collected via lumbar puncture (LP) with CSF total nucleated cells (TNC) <5, low glucose (<40 mg/dL), or abnormal protein (<15 mg/dL or <45 mg/dL) in non-neutropenic adults. Our electronic medical record (EMR) also provides hard stops if order indication for ME panels include “Evaluate for ventriculoperitoneal shunt infection” or “Evaluate for brain mass/abcess.” An “other” option was made available to prompt an infectious disease consult to discuss clinical indication for the test. **Results:** Twenty-three ME panels were performed on adult inpatients and outpatients at our healthcare system over the month reviewed. All 23 ME panels resulted as normal for all 14 pathogens. Eleven out of 23 ME panels met clinical indication outlined in our microbiology lab’s diagnostic stewardship algorithm. Ten out of 23 were ordered for meningitis in an immunocompromised host, although none of the patients had WBC <1 or ANC <0.5. Five out of 10 panels were ordered on immunocompromised individuals who did not were not appear immunocompromised upon chart review. Two out of six ME panels were run for community acquired meningitis despite completely normal CSF parameters. **Conclusion:** There is a discrepancy between our institution’s diagnostic stewardship algorithm indications for for ME panels and actual ME panel ordering and completion. There is also a high rate of negative ME panel test results which may indicate test overuse. Re-evaluation of our ME panel algorithm and the utility of a clinical decision rule are warranted.

